# Need-standardised comparisons must not become a barrier to recognising and addressing sex and gender disparities in major trauma care

**DOI:** 10.1186/s13049-026-01652-y

**Published:** 2026-06-23

**Authors:** Francois-Xavier Ageron, Tim Nutbeam, Amandine Ghika-Nanchen, Perrine Truong, Pierre-Nicolas Carron, Carole Clair, Angèle Gayet-Ageron

**Affiliations:** 1 Emergency Department, Lausanne University Hospital, Bugnon 46, Lausanne, 1011 Switzerland; 2 University Hospitals Plymouth NHS Trust, Plymouth, UK; 3 Department of Ambulatory Care, Unisanté, Lausanne, Switzerland; 4 Institute of Social and Preventive Medicine, University of Bern, Bern, Switzerland

We read with interest the matters arising by Fouche regarding the scoping review by Ghika-Nanchen et al. [1, 2] The author calls for more evidence and need-standardized comparisons before drawing conclusions about gender disparities in trauma care [1]. He highlights methodological limitations in the studies included in the review, with which we agree. However, all these limitations were already acknowledged in the scoping review [2]. 

The author argues that healthcare disparities and treatment differences can only be demonstrated after patients are made comparable based on ‘legitimate clinical need, treatment preference, and the appropriateness of the intervention’. He advocates for using a comprehensive set of adjustment variables to account for all potential confounders and states that these variables are either absent or poorly measured, making it impossible to assess any differences [1]. This argument is theoretically sound. However, it rests on an assumption that clinical need can be assessed independently of the sex and gender effects under investigation. In major trauma triage, that assumption deserves scrutiny. If clinicians systematically underestimate injury severity in women presenting with pelvic fractures, low-energy mechanisms, or spinal injuries that are more difficult to identify at scene [3], then triage priority and transport decision are not clean measures of clinical need available for neutral adjustment: they may partly express the disparity itself. Adjusting for them would not achieve comparability but would instead remove the mechanism of interest from the analysis. The relevant question is therefore not only whether women received less care given their assessed need, but whether their need was assessed equitably in the first place.

Pre-hospital clinicians have to make difficult decisions under uncertainty within a few minutes. These conditions often lead to the application of stereotypes with implicit bias. This matters for how bias itself is defined. Fouche’s framework implicitly requires that a sex or gender coefficient survive full clinical need-standardisation before it can be characterised as bias or discrimination. But implicit bias, as understood in both psychological and health equity literature, operates precisely through pattern recognition and heuristic reasoning under time pressure. It does not require intent. It does not require a residual coefficient in a fully adjusted model. Under the standard epidemiological definition of health disparity, a difference in treatment that is not attributable to appropriate clinical variation in need constitutes a disparity regardless of whether discrimination was conscious. In this context, the use of confounders that themselves encode implicit bias is a key problem. Including them will bias results toward the null rather than achieve comparability. The Injury Severity Score (ISS) is probably the least biased adjustment variable, as it is based solely on anatomical description of injury. Although it cannot be calculated at the scene, it reflects an objective measure of injury severity at baseline, allowing for meaningful comparison.

The circumstances of injuries differ by gender, reflecting risky behaviours in young men. However, women are still victims of severe injuries independently of the mechanism of injury. Women are more likely to suffer from pelvic and spine injuries, which are more difficult to identify at the scene [3]. Consequently, a young man stabbed with no signs of haemodynamic instability and without life-threatening injury is more likely to be transported to a trauma centre than a middle-aged woman with an unstable pelvic ring fracture. Adjusting for confounders that themselves carry some degree of implicit bias risks removing the disparity from the model rather than controlling for it. The appropriate approach is not to maximise the number of adjustment variables but to identify, using a directed acyclic graph (DAG), those variables that affect both the exposure and the outcome without lying on the causal pathway between them [4]. Moreover, including covariates that are mediators or colliders rather than true confounders will remove the direct effect of sex/gender from the total effect estimate, leading to a false interpretation of model coefficients [5]. In practice, a DAG-guided approach, rather than inclusion of every conceivable variable, provides the appropriate methodological framework for this question.

We agree with Fouche that causality cannot be demonstrated in observational studies and that routine-care registry data may introduce bias. While residual confounding is possible, the repetition of similar findings across different studies and settings strongly suggests the existence of gender disparities. For example, the study by Nutbeam et al. reported a clear gender disparity in tranexamic acid administration, using appropriate adjustments for injury severity, bleeding risk, and treatment eligibility [6]. Limiting the scope of these findings to a simple lack of need-standardized comparisons does not address the core issue and perpetuating gender disparities.

We recognise that studies assessing gender bias may lead to the concept of “uncomfortable knowledge,” as described by Rayner [7]. Attempting to find explanations to account for observed disparities is a natural scientific response to these findings. This review did not aim to assign blame; rather, it sought to report and quantify the disparities observed. The question is not to limit the debate to semantic distinctions between sex differences understood in a narrowly biological sense and gender disparities understood only as intentional discrimination. Bias, as the epidemiological and psychological literature defines it, encompasses structural and cognitive mechanisms that operate without conscious intent. Recognising that disparities exist, and that their mechanisms may be modifiable, is the starting point for improvement.

We agree that need-standardised comparisons are important for future studies, and we share Fouche’s call for prospective, sex-disaggregated data collection with careful specification of the decision point and adjustment set. However, the evidential standard required should be proportionate to the plausibility and consistency of the findings, not so demanding that it precludes action where the signal is already coherent across multiple settings and study designs.

Need-standardised comparisons matter, and future research should pursue them. But the current evidence, considered with appropriate caution, is already sufficient to justify system-level attention to how women are triaged, transferred, and treated in major trauma. The priority now is to develop and validate interventions and tools that provide objective triage and treatment criteria for all.

## Data Availability

No datasets were generated or analysed during the current study.
